# The prospect of rising in rank is key to long-term stability in Tibetan macaque society

**DOI:** 10.1038/s41598-017-07067-1

**Published:** 2017-08-01

**Authors:** Lixing Sun, Dong-Po Xia, Shine Sun, Lori K. Sheeran, Jin-Hua Li

**Affiliations:** 1 Department of Biological Sciences, Central Washington University, Ellensburg, Washington, 98926 United States of America; 2 School of Life Sciences, Anhui University, Hefei, P.R. China; 3 Central Washington University/EHS, Ellensburg, Washington, 98926 United States of America; 4 Department of Anthropology, Central Washington University, Ellensburg Washington, 98926 United States of America; 5 School of Resource and Environmental Engineering, Anhui University, Hefei, P.R. China

**Keywords:** Behavioural ecology, Animal behaviour

## Abstract

One of the most fundamental questions in behavioural biology is why societies can persist for a long period of time. While researchers in animal behaviour have been hindered by a lack of an aggregate measure (such as social mobility) to quantify the dynamics of animal societies, researchers in social sciences have been challenged by the complexity and diversity of human societies. As a result, direct empirical evidence is still lacking for the hypothesized causal relationship between social mobility and social stability. Here we attempt to fill the void by examining a much simpler society in the Tibetan macaque (*Macaca thibetana*), which we have tracked for 30 consecutive years. By testing two group-level hypotheses based on benefit-cost analysis and social stratification, we show the first quantitative evidence that an annual 2-to-1 stay/change ratio in the hierarchy with a 3-to-1 upward/downward ratio in intragenerational social mobility provides a substantive expected benefit for adult members to stay in the group and wait for their chances to advance. Furthermore, using a Markov transition matrix constructed from empirical data, we demonstrate that the 3-to-1 upward/downward ratio could lead to long-term structural stability in Tibetan macaque society.

## Introduction

One of the most fundamental yet challenging questions in the study of animal behaviour and social evolution is why many animal societies can persist for a long period of time. Why, phrasing it differently, do many social animals choose to stay in the same groups for years? This question has been traditionally addressed by arguing that social living brings more benefit (such as a reduced predation risk) than cost (such as higher rates of parasites and diseases and intensified competition)^1^. However, such benefit-cost analyses, while presenting a strong adaptive logic in the choice of behaviour for individuals, do not directly answer why a particular society can persist in the long run. In any hierarchical society, in fact, high-ranking members generally have better access to resources critical for survival and reproduction (such as food and mates) than do low-ranking members^2–4^. This means that many low-ranking members, compared with their high-ranking peers, suffer a relative fitness loss^5^. Why would they still stay in the same group for years? A well-known reason is that dispersal (emigration) and switching groups may incur a higher cost^6, 7^. An equally important, yet rarely considered, factor that can compel members to stay in the group, however, lies in the prospect to advance in the hierarchy, especially when no better alternative exists. As such, social mobility, defined as the process by which individuals move from one status position to another within a society^8^, is broadly believed to be essential for social stability among social scientists.

First noticed by Alexis de Tocqueville in the 19^th^ century^9^, social mobility today is widely believed to play a key role in economic development, equality, social justice, and public health in human societies, for which it is of great interest to researchers across a broad range of social and natural science disciplines^10–14^. Even so, how social mobility is related to social stability is still unclear owing to a lack of direct empirical evidence^15^. This situation is mainly due to the fact that human societies, especially modern human societies, tend to be far too large in size, too complex in structure, and too diverse in culture to find a clear relationship between social mobility and social stability^8, 15, 16^.

Obviously, social mobility, as it is defined, is not unique to humans but a feature of all animals that live in relatively stable, hierarchical groups. In fact, researchers in animal behaviour have developed an array of innovative, discipline-specific methods (such as using spacing behaviour and the Elo-rating method) to understand the relationships among socioecological factors, dominance hierarchy, and social stability^17–20^. It is unfortunate that an interdisciplinary barrier has hampered free information exchange between social and biological sciences, resulting in a situation where researchers in animal behaviour somehow overlook the importance of social mobility on the one hand, whereas researchers in social sciences are challenged by the complexity and diversity of human society on the other. One logical resolution to this issue is to introduce intragenerational social mobility (social mobility or mobility, thereafter), an aggregate indicator broadly used in social sciences to gauge lifetime opportunities for social advancement, into the study of smaller and simpler animal societies.

The usually much smaller and simpler nonhuman primate societies are well suited for such a cross-disciplinary approach, not only because they can allow us to circumvent the structural complexity and cultural diversity of human societies but also because they can provide new insights into the evolutionary connection in social dynamics between humans and other animals. The Tibetan macaque (*Macaca thibetana*), in particular, is one of the best primate subjects to investigate the relationship between social mobility and social stability for several reasons. First, all adults of the species live in a small, matrilineally-structured group with a strictly linear hierarchy^21, 22^, which is simple even when compared with many other macaque species living in larger groups with more complex social relationships^23^. Also, their dominance hierarchy usually remains stable for several months or more, which facilitates observation and unambiguous determination of social status at regular intervals (e.g. annually, as in this study) for every adult in the group. Moreover, we have tracked one particular group of the species for 30 consecutive years, which provides a substantial dataset for analysing the dynamics of its hierarchy over time. Finally, the group has experienced four permanent fission events over the 30-year period (unpublished data), which indicate a group-level process that can perpetuate the long-term sustainability of the macaque society in the wild.

Because social mobility reflects lifetime opportunities for social advancement, it is likely to play a key role in the coherence and perpetuation of the macaque society. Moreover, since social stability is a society-level phenomenon, it contains emergent properties that may not be easily understood from the summation of individual behaviour^24, 25^. Therefore, investigating social mobility, an aggregate measure of the group, may be more likely to lead to new insights into social stability than studying any individual-based behaviour. Accordingly, we here propose two novel group-level hypotheses to examine how social mobility is related to social stability: 1) from the perspective of an aggregate benefit/cost ratio for the entire group, the upward mobility should exceed the downward mobility, and 2) from the perspective of social structure, the level of mobility existing in the macaque society should drive the social stratification process to a stable state in the long run. Hypothesis 1 specifies an overall incentive condition for all group members to stay in, rather than leave, the group. Hypothesis 2 stipulates that a mobility-driven group process can converge to a stable state in social stratification over time, essential for the long-term stability of a society^26^. In this study, we used social mobility to test hypothesis 1 by performing a group-level benefit-cost analysis based on the probability of upward/downward movement in the hierarchy and hypothesis 2 by probing into the long-term structural dynamics of the macaque society using a Markov model.

## Results

**Figure 1:**
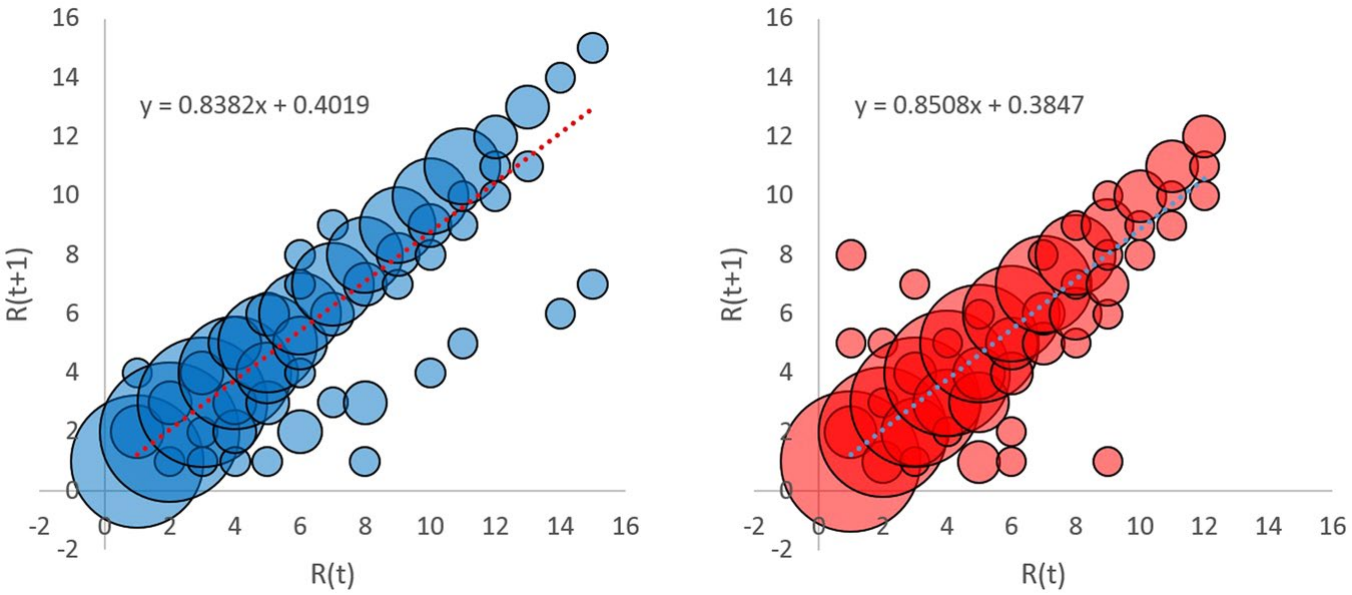
Social mobility in terms of rank at time t (R(t)) and rank a year later (R(t+1)) for the same individuals (left: males, N = 182 individual-years; right: females, N = 192 individual-years). The regression equation for the dotted line in each panel shows the relationship in rank between two consecutive years. Note the number of overlapping data points is proportional to the size of the bubble.

The annual rate of social mobility in the Tibetan macaque society was 0.1618 (r = 0.9012, df = 181, P = 0.000) for males and 0.1482 for females (r = 0.8906, df = 191, P = 0.000, Fig. 1). Test for the slopes of the regression lines showed no difference between the sexes. Residue analysis revealed a shortage of extreme values predicted by normal distribution (Anderson-Darling test, AD = 11.198, N = 182, *p* < 0.005 for males; AD = 8.348, *n* = 192, P < 0.005 for females), indicating that large changes in rank (≥3) in a year tended to be rare (22.03% for males out of N = 59 total changes observed and 18.03% for females out of N = 61 total changes observed) whereas most changes were within two ranks (77.97% for males and 81.97% for females for all changes).

**Figure 2:**
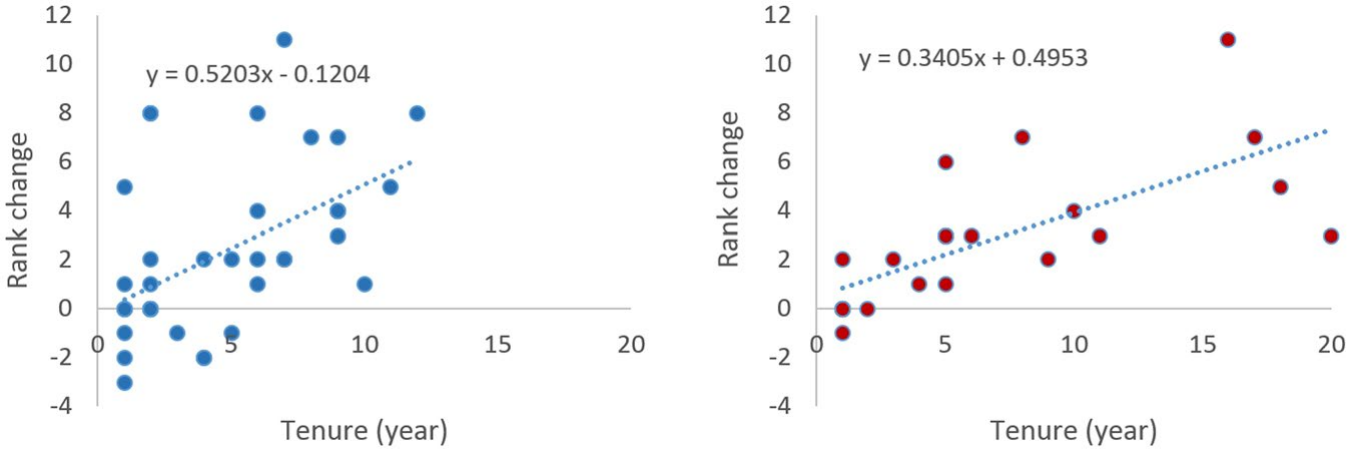
Relationship between tenure in the group and rank change (left: males; right: females). Tenure refers to the entire duration (number of years) an individual stays in the group. The regression equation for the dotted line in each panel shows the relationship in rank between tenure and rank change.

Over time, the probability of upward mobility rose with an adult’s tenure in the group (Table 1), which in turn was positively correlated with the degree of upward change in rank (r = 0.5276, df = 38, P = 0.001 for males; r = 0.7650, df = 23, P = 0.000 for females, Fig. 2), However, a Kaplan-Meier survival analysis failed to show a significant difference between males and females in upward mobility or staying at the same ranks. Our analysis could not be applied to downward mobility because seven males but only one female moved downward. Cumulatively, social mobility could lead to large degrees of flux in the hierarchy with the unbroken tenure in the group being only 2.89 (SD = 2.42, N = 9) years for alpha males, 4.33 (SD = 2.16, N = 6) years for alpha females, 1.27 (SD = 0.63, N = 22) years for omega males, and 1.53 (SD = 1.07, N = 17) years for omega females. However, Mann-Whitney U tests failed to show a significant difference between the sexes at both the top (U = 61.5, P = 0.2288) and the bottom (U = 419.0, P = 0.4306) of the hierarchy.

**Table 1:** Tenure in the group in relation to social mobility.

Tenure (years)	Probability (male)			Probability (female)		
	Upward	Downward	No Change	Upward	Downward	No Change
≥1	0.6154	0.1795	0.2051	0.7083	0.0417	0.2500
≥2	0.7097	0.1290	0.1613	0.9444	0.0000	0.0556
≥3	0.8182	0.1818	0.0000	1.0000	0.0000	0.0000
≥4	0.8571	0.1429	0.0000	1.0000	0.0000	0.0000
≥5	0.9444	0.0556	0.0000	1.0000	0.0000	0.0000
≥6	1.0000	0.0000	0.0000	1.0000	0.0000	0.0000

**Figure 3:**
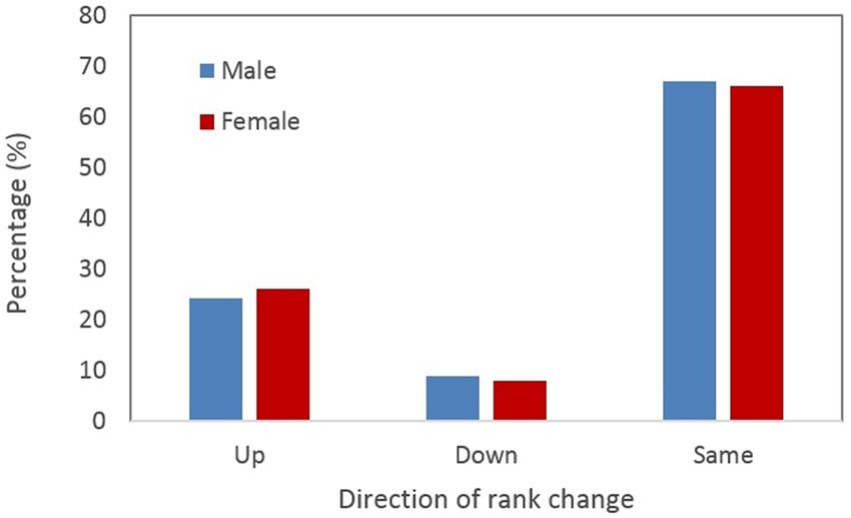
Percentage (%) of rank changes between two consecutive years (‘Up’ = upward change; ‘Down’ = downward change; ‘Same’ = no change).

For both sexes between two consecutive years, ranks were mostly stable with a probability of 0.666 for no-change in rank, twice as high as the probability of 0.334 for change (*χ*^*2*^ = 44.281, df = 1, P = 0.000, assuming equal probability for change versus no-change). When ranks did change, the probability of upward mobility (0.251) was three times as high as that of downward mobility (0.083, *χ*^*2*^ = 31.752, df = 1, P = 0.000). Again, we found no difference between the sexes (Fig. 3).

Using yearly data, we constructed Markov transition matrices between upper and lower strata of the hierarchy for males, females, and combined sexes, respectively:

$$[\begin{array}{cc}H-H & H-L\\ L-H & L-L\end{array}]=[\begin{array}{cc}0.9570 & 0.0430\\ 0.1854 & 0.8146\end{array}],[\begin{array}{cc}0.9184 & 0.0816\\ 0.1985 & 0.8015\end{array}],{and}\,[\begin{array}{cc}0.9375 & 0.0625\\ 0.1922 & 0.8078\end{array}],$$

where H–H, H–L, L–H, and L–L represent probabilities of transition from high to high (stay), high to low (downward change), low to high (upward change), and low to low (stay) within or between the two strata of the hierarchy. We estimated the odds ratio of an adult ending its life in the upper and lower strata (Table 2). Males apparently had a higher probability of ending in the top half of the hierarchies than females. When the sexes were combined, the ratio converged at approximately 3/1, the limiting probabilities for the Markov transition matrix.

**Table 2:** Social stratification process over time (year) driven by upward/downward mobility (values presented as probabilities) for males, females, and combined sexes.

Number of Years	Male (H-H/L-L)	Female (H-H/L-L)	Combined (H-H/L-L)
2	0.9238/0.6715	0.8597/0.6586	0.8909/0.6646
4	0.8784/0.4759	0.7870/0.4817	0.8303/0.4783
8	0.8353/0.2902	0.7298/0.3424	0.7779/0.3173
16	0.8146/0.2011	0.7107/0.2949	0.7568/0.2523
32	0.8117/0.1886	0.7088/0.2912	0.7546/0.2455

A Markov process stabilizes when the two diagonal elements (H-H and L-L) asymptotically approach certain values (limiting probabilities). By our operational definition (see the Methods section), the limiting probabilities are reached between 16 and 32 years, approximately the lifespan for males and females as adult.

## Discussion

Using social mobility to quantify social opportunities in this study, we show that stability was the dominant pattern in Tibetan macaque society with two-thirds of adults staying at the same ranks and one-third changing their ranks annually. Social stability was also demonstrated by the rarity of large annual changes in rank over our 30-year study period, a result similar to the pattern observed in other primates such as rhesus macaques, *M. mulatta*^27^.

Because Tibetan macaques tend to be despotic in dominance style^28^, the risk of ‘queue-jumping’ in the hierarchy should be high as observed in savannah baboons, *Papio cynocephalus*^29^. In contrast, with a considerable level of cumulative mobility over time (Fig. 2) and the 3-to-1 odds favouring upward movement, waiting for opportunities by staying in the group was an adaptive strategy because of the large expected net gain for all adults in the group from our social mobility analysis. Therefore, our first hypothesis that the overall benefit should exceed cost at the group level is supported. It is likely that waiting may be a better strategy for social advancement than leaving the group and finding opportunities elsewhere. This proposition, though requires empirical tests, has a good parallel in another ‘despotic’ species, the rhesus macaque, where males follow a ‘seniority rule’ and wait in queue for a rise in rank^30^.

Because social mobility is an aggregate measure, a gauge of expected (or average) opportunities for all adults in the entire group, and low-ranking members have more upward potentials than high-ranking members, the expected net benefit of waiting should present a larger incentive for low-ranking members than for high-ranking members. Thus, a larger future prospect for social advancement may partly offset the current lower status in the group, making low-ranking members more incentivized to stay in than leave the group. Additionally, the predominance of small rank change over time might also remove some uncertainty associated with mobility. These conditions favouring members (especially low-ranking members) to stay may explain why our macaque group could persist for three decades.

In testing hypothesis 2 about the social stratification process, our use of the two-stratum classification system was both strategical and practical due to small group size (see Methods). From the strategical perspective, if the average rank represents a demarcation between advantage and disadvantage in relative fitness^5^, pooling individuals into two equal strata has a clear evolutionary implication for the behavioural strategy of waiting: those in the upper stratum should be advantageous whereas those in the lower stratum should be disadvantageous when compared with the average fitness (because of the roughly linear relationship between rank and fitness in Tibetan macaque groups). As such, for individuals in the lower stratum, waiting reflects a trade-off in relative fitness between the present (low) and the future (high). It will not be an adaptive strategy unless the net upward mobility is sufficient.

Modelling social stratification using the Markov process allowed us to further explore the long-term structural dynamics of Tibetan macaque society^31, 32^. From a mathematical standpoint, since not all transition matrices have limiting probabilities, the very existence of limiting probabilities in the transition matrices constructed from our empirical data may present another reason for why the group could persist for 30 years. Clearly, such an outcome was a result of the mobility in the group. In other words, the 2-to-1 change/stay ratio and the 3-to-1 upward/downward ratio could drive the stratification process to approach a stable state from dynamic transitions between the upper and lower strata of the hierarchy. As such, hypothesis 2 is also supported. The asymmetrical upward/downward ratio was due mainly to deaths and departures of especially high-ranking group members^21^. Such changes are commonly observed in primates^7, 29^ and are comparable to retirement, demotion, and job change of high-ranking members in human organizations^10, 26^.

Also noticeable was the rate of the three transition matrices approaching their limiting probabilities (between 16 to 32 years, Table 2), which approximated the lifespan (14–28 years) of males and females as adult in this species (unpublished data). Although males appeared to have a higher probability to end up in the upper half of the hierarchy within their lifetimes, females might offset this disadvantage by reaching sexual maturity two years earlier^21^. As in the majority of primates^33–38^, social status is positively related to fitness (especially for males) in Tibetan macaques^21^. Here, upward social mobility becomes essential for social stability because, without the prospect to rise above the average in rank (and in fitness), low-ranking members may not be incentivized to stay in the group. From the long-term social stratification point of view, the favourable 3-to-1 odds ending in the upper versus lower stratum in macaque society appear to be adequate to motivate individual members to stay. That is, by adopting the strategy of waiting for social advancement opportunities, they would have a good chance to achieve above-average social status and above-average fitness in their lifetimes. This may explain why the macaque society could perpetuate for decades.

It was surprising that we did not detect any difference in social mobility between the sexes. Sexual selection is intense in many primates including Tibetan macaques^21, 34^, where low-ranking males can pose a strong pressure against social stability^5^, leading to a less stable male hierarchy. Indeed, in many matrilineally-organized primates such as macaques and baboons, rank change is more frequent in males than in females^39–42^. Apparently, one way to stabilize the male hierarchy is through behavioural regulation (e.g., aggression, reconciliation, peace-making, policing, food-sharing, and grooming) by high-ranking males^43–46^. This, as a result, may reduce sexual difference in social mobility. Furthermore, since several factors such as kinship^47^, social connectivity^48–50^, coalition formation^48, 51^, age^29, 52^, and personality^53, 54^ can play important roles in determining dominance rank in primate societies, they may also affect social mobility. These important factors should be closely and systematically scrutinized in the future.

In conclusion, by quantifying lifetime opportunities with the aggregate measure of social mobility, we were able to use a group-level benefit-cost analysis and Markov modelling to answer why Tibetan macaque society could sustain for decades from two different perspectives: expected fitness benefit for all adult group members and structural dynamics of the entire hierarchy. These mobility-centred approaches not only allowed us to find the opportunity incentives that could motivate group members (especially low-ranking members) to stay in the group but also open up a new way of quantitative investigation on the dynamics of animal societies as a whole.

## Methods

Our study subject, the Tibetan macaque, lives in small, matrilineally-organized groups, where females (sexually mature ~5 years) stay for life and males typically disperse at sexual maturity (~7 years)^55^. In this study, we collected data from a group of free-ranging macaques for 30 consequetive years since 1986 at Mt. Huangshan, a natural reserve located in Anhui Province, China. To facilitate research and attract tourists, our study group, known as YA1, was provisioned with a total of 3–4 kg of corn a day^50^. Provisioning occurred every day at 09:00, 11:00, 15:00, and 17:00. When corn was depleted after about 30 minutes, the macaques left the provisioning area and continued their activity in the forest nearby. No evidence was found that provisioning and other anthropogenic factors (such as the occurance of park rangers and tourists) affected the stability of hierarchy when we compared data from YA1 and from another group (YA2) in the vicity, which was much less affected by humans. Detailed background information about the study species and the biotic and abiotic conditions about our study location such as climate, vegetation, predator condition, management practice, tourist flow, and group history has been reported elsewhere^50, 55–59^. Here we focus on presenting information specific to testing the two hypotheses in this study.

We observed the group for a total of 5–10 months each year and identified all group members based on their distinctive facial or body features and mapped out their dominance relationships based on aggressive-submissive interactions scored *ad libitum* (see Supplementary information for further detail). Aggressive interactions were defined as an individual threatening, chasing, slapping, grabbing, or biting another individual. Submissive behaviors were scored when an individual showed fearful interactions, such as fear grin, cower, mock leave, avoid, flee, or scream^28^. All records of agonism were tallied and divided into aggression received^28^ and aggression given for each adult dyad. We then created a win:loss matrix that summarized for all dyads the tallies of agonistic interactions that included an aggressive act followed by a submissive response. According to the method detailed in Martin and Bateson^60^, we derived a rank order by re-arranging the matrix to minimize the number of reversals. Despite potential problems in this method^61^, we rarely encounter any ambiguity in assigning rank due to a large amount of data collected, clear linearity, and relative stability in the dominance order of our study group^21^. More details about how we recognized individuals, defined behavioural patterns, compiled data, and constructed dominance hierarchy are available^22, 28^.

In the current study, due to age difference in sexual maturation as also seen in other primates, we analysed adults that were seven years or older to control for the effect of age on dominance between young males and females^52, 62–64^. (Female were born in the group and their ages were positively known. Males were immigrated from other groups and their ages were estimated, typically within 2 years as the margin of error when compared with males of known ages.) We use the word ‘tenure’ for the entire duration (number of years) for an individual in the group as adult (≥7 years). For comparisons between the sexes, we kept two separate hierarchies for males and females.

Since social mobility has never been measured in nonhuman animals, we adopted two quantitative methods commonly used in the study of human societies: mobility over time for the cumulative effect and the rate of mobility for change per time period (year in our case)^16^. Social mobility can be measured in terms of absolute change in rank (that is, change in rank without referencing to others in a hierarchy) or relative change in rank (that is, change in rank relative to others’ ranks in a hierarchy, commonly expressed as change in percentile^26^). Both measurements have their strengths and weaknesses. Preliminary analysis in our study showed that the use of relative mobility resulted in a much larger variation and sometimes inconsistency in rank change. At times, an increase in absolute rank might lead to a decline in relative rank due to fluctuations in group size (mean = 15.79 adults, SD = 6.06). (For example, the third-ranking individual would be ranked differently in relative rank in a group of 12 versus a group of 20). To avoid this problem, we used absolute mobility in measuring the cumulative effect to test hypothesis 1 while leaving relative mobility for the group stratification process when we explored structural dynamics of the group over time to test hypothesis 2.

We define social stability as the persistence of a hierarchy without change over time. Thus, the rate of mobility can be measured by 1 - b, where b is the slope of the regression line for rank change from time t to time t+1^16^. As such, each sample point was an individual-year. We assessed each regression line by testing the significance of Pearson’s correlation coefficient r. To analyze the difference between the sexes, we compared the slopes of the regression lines between males and females using t-test^65^.

We tested hypothesis 2 by investigating whether the current level of social mobility could lead to a long-term stable state between the strata of the hierarchy. To do so, we constructed a Markov transition matrix and found its limiting probabilities^31^. To construct Markov transition matrices, we divided all adults in our study group into the upper and lower strata of equal size, contrary to human societies, which are often sliced into strata by the quantile or 20% increment (see the Discussion section for the reasons)^66^. If a group had an odd number of adults of either sex, the middle-ranking individual was given two half-scores for the upper and the lower strata, respectively. We then constructed Markov transition matrices with the probabilities of change and no-change in rank on a yearly basis and then found the limiting probabilities by exponentially multiplying the transition matrices until the values stabilized at 0.001 level. Other important statistics are presented as they occur in the Results section. All statistics were conducted using SPSS (Version 24) or Minitab (Version 17), and all tests were two-tailed with the alpha value set a priori at 0.05.

All research protocols reported here were approved by the Institutional Animal Care and Use Committee of Central Washington University and the Chinese Wildlife Management Authority. All of the research work complied with the Wildlife Protection Law of the People’s Republic of China, the regulatory requirements of Huangshan Garden Forest Bureau, China, where the study took place, and the American Society of Primatologists’ ethical standards.

### Ethical considerations

The Chinese Wildlife Conservation Athority approved the research, which also adhered to the American Society of Primatologists’ ethical standards.

## Electronic supplementary material

Supplementary information
