# Publisher Correction: The prospect of rising in rank is key to long-term stability in Tibetan macaque society

**DOI:** 10.1038/s41598-020-68787-5

**Published:** 2020-07-24

**Authors:** Lixing Sun, Dong-Po Xia, Shine Sun, Lori K. Sheeran, Jin-Hua Li

**Affiliations:** 1 Department of Biological Sciences, Central Washington University, Ellensburg, Washington 98926 United States of America; 2 School of Life Sciences, Anhui University, Hefei, People’s Republic of China; 3 Central Washington University/EHS, Ellensburg, Washington 98926 United States of America; 4 Department of Anthropology, Central Washington University, Ellensburg, Washington 98926 United States of America; 5 School of Resource and Environmental Engineering, Anhui University, Hefei, People’s Republic of China

Correction to: *Scientific Reports* https://doi.org/10.1038/s41598-017-07067-1, published online 01 August 2017

In the original version of this Article, Dong-Po Xia was incorrectly listed as a corresponding author. The correct corresponding authors for this Article are Lixing Sun and Jin-Hua Li. Correspondence and request for materials should be addressed to lixing@cwu.edu or jhli@ahu.edu.cn.

Additionally, the original version of this Article contained an error in Affiliation 1 which was incorrectly given as ‘Departmenet of Biological Sciences, Central Washington University, Ellensburg, Washington, 98926, United States of America’. The correct affiliation is given below:

Department of Biological Sciences, Central Washington University, Ellensburg, Washington, 98926, United States of America.

These errors have now been corrected in the HTML and PDF versions of the Article, and in the accompanying Supplemental Material.

