# Supplementary material for: The prospect of rising in rank is key to long-term stability in Tibetan macaque society
Source: Sci Rep. 2017 Aug 1;7:7082. doi: 10.1038/s41598-017-07067-1 (PMC5539219; doi:10.1038/s41598-017-07067-1)
Supplement: Supplementary file 1 — Supplementary information [file 41598_2017_7067_MOESM1_ESM.pdf]

The prospect of rising in rank is key to long-term stability in Tibetan macaque society

Lixing Sun[1,*], Dong-Po Xia[2,*], Shine Sun[3], Lori K. Sheeran[4], Jin-Hua Li[5]

[1]Department of Biological Sciences, Central Washington University, Ellensburg, Washington 98926, United States of America. [2]School of Life Sciences, Anhui University, Hefei, P.R. China. [3]Central Washington University/EHS, Ellensburg, Washington 98926, United States of America. [4]Department of Anthropology, Central Washington University, Ellensburg, Washington 98926, United States of America. [5]School of Resource and Environmental Engineering, Anhui University, P.R. China. Correspondence and requests for materials should be addressed to L.S. (email: lixing@cwu.edu) or J.-H.L. (email: jhli@ahu.edu.cn)

*co-first authors

Supplementary information:

Complete dataset about the ranks of adult Tibetan macaques of the YA1 group (1987 – 2015). Note: the rank orders were constructed based on a total of 5-10 months of observation (scattering over 6-12 months of field work) per year. The individuals included were all adult members (see the definition in the Methods section in the article) that were present in the group for at least 6 months of the year.

Males:

**Name**	87	88	89	90	91	92	93	94	95	96	97	98	99	00	01	02	03	04	05	06	07	08	09	10	11	12	13	14	15
Daqiang	1	1																											
Huangmao	2	2	1	1	1	1	1																						
Zuoshizhi	3	3																											
Wushang	4	4	2	2	2																								
Chazui	5	6																											
Erxiong	6	7	3	3	3	2	2																						
Kelian		8																											
Youshizhi		5	4	4																									
Chiyan			5	5	4	3	3	3	3	3																			
Yanbaiban			6	6	5	4	4	4	4																				
Duyanlong					7	9	9	7	7																				
Huangzhong					6	5	5	5	5	4	5	5	5	5	5	1													
Sanxiong						10	10	8	8	7	6	6	6	6	6	2	2	2											
Yelai						11	11	9	9	8	7	7	7	7	7														
Xingxiong						6	6	2	2	2	2	2	2	2	2														
Gaoshan						7	7	6	6	5	3	3	3	3	3				3	3	4	2	2	3	3	3	3	4	5
Gaoxiong						8	8	1	1	1	1	1	1	1	1														
Zhaoyun							12																						
Chawei								10	10	9	8	8	8	8	8	3													
Yaqiang								11	11	10	9	9	9	9	9														
Zhifeng									12	11																			
Huagang										12	10	10	10	10	10	4	1												
Zhiming										13	11	11	11	11	11	5													
Heipi										6	4	4	4	4	4														
Zhibing													12	12	12														
Tengben													13	13	13														
Zhilong														14	14	6													
Tengyuan														15	15	7													
Huaheng															16		4												
Panpan																8	3												
Huayong																	5												
Hualang																		1	2	2	2								
Yeda																			1	1	1								

Zilong	4	3	1	1	2	2	2	2	3	3
Yetian		6								
Huanggai		5	3	3						
Changmao			4	4	4					
Santui			5	5	5					
Changtong			6							
Tougui					1	1	1	1	2	2
Baitou						4	4	4	5	6
Zuoba									1	4
Duanshou									6	8
Heitou									7	7
Yerongbing										1

Females:

**Name**	87	88	89	90	91	92	93	94	95	96	97	98	99	00	01	02	03	04	05	06	07	08	09	10	11	12	13	14	15
Gen	1	1																											
Gan	2	2																											
Zhi	3	3	1	1	1	1	1	1	1	2																			
Baijiamao	4	4	2	2																									
Ye	5	5	3	3	2	2	2																						
Ya	6	6																											
Shi	7	7																											
Hua				4	3	3	3	2	2	3	2	2	1	1	1	1	1	5	1	1	1	1	1	1					
Teng					4	4	4	3	3	4																			
Changlian						7	7	5	5	6	4	4	4	4	4	3													
Feimao						5	5	4	4	5	3	3	3	3	3	2	2												
Beihen							8	6	6	1	1	1	2	2	2														
Tougou							9	7	7	8	5	5	5	5	5	4	3	4	4	4	4	4	4	4					
Eshang								8	8	9	6	6	6	6	6	5	4												
Huarui											8	8	8	8	8	7													
Toutai												9	9	9	9	8	6	2	5	5	5	5	5	5	3	3	3	3	7
Feitian												10	10	10	10	9	7												
Yemai											7	7	7	7	7	6	5	3	3	3	3	3	3	3	2	2	2	2	2
Beixiao												11	11	11	11	10	8												

Yezhen								12	12	12	11	9	1	2	2	2	2	2	2	1	1	1	1	8
Feiyan											12	10												
Touhong																			6	4	4	4	4	3
Yehong																			7	5	5	5	5	1
Huahong																			8	6	6	6	6	5
Tourui																				7	7	7	7	6
Huahui																					8	8	8	
Touxiaxue																								4
zhican	6	6																						
Baiyan				9	10																			
Baixiang																								
Zhifeng					7																			
